# Cost of Transport of Undulating Fin Propulsion

**DOI:** 10.3390/biomimetics8020214

**Published:** 2023-05-23

**Authors:** Tim G. A. Vercruyssen, Sebastian Henrion, Ulrike K. Müller, Johan L. van Leeuwen, Frans C. T. van der Helm

**Affiliations:** 1 ExRobotics, Delftechpark, 2629 HS Delft, The Netherlands; vercruyssentim@gmail.com; 2 Corporate Research and Development, Royal Boskalis, 3356 LK Papendrecht, The Netherlands; sebastian.henrion@gmail.com; 3 Department of Biology, Fresno State University, Fresno, CA 93740, USA; 4 Experimental Zoology Group, Department of Animal Sciences, Wageningen University, 6700 AJ Wageningen, The Netherlands; johan.vanleeuwen@wur.nl; 5 Biomechatronics and Bio-Robotics, Delft University of Technology, 2629 HS Delft, The Netherlands

**Keywords:** undulatory fins, robotic underwater vehicle, finned swimming, cost of transport, biomimetic robot

## Abstract

Autonomous robots are used to inspect, repair and maintain underwater assets. These tasks require energy-efficient robots, including efficient movement to extend available operational time. To examine the suitability of a propulsion system based on undulating fins, we built two robots with one and two fins, respectively, and conducted a parametric study for combinations of frequency, amplitude, wavenumber and fin shapes in free-swimming experiments, measuring steady-state swimming speed, power consumption and cost of transport. The following trends emerged for both robots. Swimming speed was more strongly affected by frequency than amplitude across the examined wavenumbers and fin heights. Power consumption was sensitive to frequency at low wavenumbers, and increasingly sensitive to amplitude at high wavenumbers. This increasing sensitivity of amplitude was more pronounced in tall rather than short fins. Cost of transport showed a complex relation with fin size and kinematics and changed drastically across the mapped parameter space. At equal fin kinematics as the single-finned robot, the double-finned robot swam slightly faster (>10%) with slightly lower power consumption (<20%) and cost of transport (<40%). Overall, the robots perform similarly to finned biological swimmers and other bio-inspired robots, but do not outperform robots with conventional propulsion systems.

## 1. Introduction

The purpose of many traditional autonomous underwater vehicles (AUVs) is primarily to gather data on large temporal and spatial survey missions at minimal operational costs (e.g., Hugin and Remus AUV, Kongsberg (Naval AUV Kongsberg, 2020); Seacat, Atlas-Electronik (Seacat Atlas Elektronik, 2020); LAUV, Oceanscan-mst (LAUV Oceanscan-mst, 2020); Marlin, Lockheed Martin (Marlin Lockheed Martin, 2020); multiple AUVs, ECA Robotics (AUVs ECA Robotics, 2020)). These commercial AUVs are designed for operational efficiency and reliability. They come in (variants of) the well-known cone-shaped, multiple-finned, single-thruster robot. This design is less suitable for more complex offshore or inshore inspection tasks around groups of oil platforms, windmill foundations, wrecks and quay walls and jetties. Such tasks require maneuvering in complex environments and variable currents or have to deal with diver presence. Hence, such tasks require good situational awareness and a high degree of maneuverability, stability and obstacle avoidance capabilities. They are typically executed by remotely operated vehicles (ROVs), and not AUVs, tethered by an umbilical to a surface ship (products such as the Schilling HD by Schilling and the Cougar TX product line by Saab Seaeye).

Aside from general design requirements for efficiency and reliability, AUVs may need to meet mission-specific demands, which creates additional design requirements and may even create design conflicts. One such additional design requirement is stealth. Intelligence missions require stealthy and silent vehicles that can avoid being spotted. Seismic data collection requires designs that minimize disturbing onboard sensors. The quality of seismic data improves when receivers of seismic signals make reliable contact with ground stations in order to avoid disturbance of the signals through the water column. Design conflicts commonly arise during operational underwater inspection missions that require more than a single well-defined task to be executed. Conventional vehicle designers struggle to build cost-effective AUVs for complex operational inspection tasks that require maneuverability in small spaces, stability for sensing and special traits such as lying on the bottom for a prolonged period of time or station-holding in current-rich environments.

Nature has a broad diversity of locomotory solutions to complex ‘operational’ requirements such as observing, foraging, mating, hiding and escaping in underwater environments, using a wide range of propulsive mechanisms and appendages. Among macroscopic species that operate in small spaces and/or close to vegetation or the seafloor, several have evolved a similar propulsive system: undulatory fins, which produce thrust by generating a transverse travelling wave along an elongated fin. Cuttlefish, squid, seahorses and knifefish are examples of animals with soft or semi-rigid bodies that use one or more undulatory fins to execute extraordinary locomotory behaviors. Sefati and colleagues showed that knifefish generate mutually opposing forces with a single undulating fin to simultaneously provide stability and maneuverability [1]. Squid and cuttlefish achieve great maneuverability due to a coordinated interplay between jetting and fin undulations [2]. The fins of squid also serve multiple purposes: generating thrust, providing lift and helping in stabilization and maneuvering [3]. These observations raise the question: given that these animals maneuver effectively in small spaces, to what extent have engineers implemented undulatory fin propulsion into highly maneuverable underwater vehicles or, even further, operational practice?

Several undulating-finned robots have been developed inspired by natural examples. Initially, robots were built to show that undulatory fins could become a viable alternative to rotational thrusters [4,5]. Once their potential was acknowledged, researchers focused on improving control, propulsion and overall design integration [6,7,8,9,10,11], as well as examining how the fins’ shape and motion affect propulsion efficiency [12], maneuverability [12,13], static thrust and swimming velocity in a flow tunnel (attaching the undulating fin mechanism to a frame with roller bearings [14]; attaching the undulating fin mechanism to a frame with air bearings [15]). Along with better robotic undulatory fin designs, researchers used quantitative flow visualization techniques on maneuvering robots (flow visualization in Supplemental Material S1 video S1, [16]) [17] or computational fluid dynamics models [18] to discover the physical principles of undulatory fin maneuvering. Recently, researchers used a robotic ribbon fin setup to verify the validity of an analytical model that claims a momentum enhancement of an undulating fin attached to a rigid flat plate [19]; analytical model explained by a series of papers by James Lighthill [20,21,22,23].

Surveyors and people responsible for inspection, repair and maintenance of underwater assets often strive for autonomous robots that can stay underwater for months or even years. This aim makes efficient energy usage and charging capabilities increasingly important. Efficient movement reduces power consumption and charging time, which extends the available operational time and enhances flexibility. Locomotion energetics are often expressed as cost of transport, which is a measure of how much power is needed to transport 1 kg of mass over 1 m or at a certain speed. Liu and Curet measured the power consumption of an undulating-fin-propelled robot. They studied 12 forward free-swimming sequences of a submerged single-finned robot at two different wavenumbers (1 and 2) and six different frequencies (0.5 to 3.0 Hz in steps of 0.5 Hz) to measure power consumption and cost of transport [24].

In this article, we characterized the power requirements of a robot swimming with an undulating fin. We conducted a parametric study to measure cost of transport for different combinations of frequency, amplitude, wavenumber and fin designs for undulatory fin propulsion. In particular, (1) we describe the design and development of a single- and double-finned free-swimming robot—the single-finned robot had four different fin designs that could be interchanged; (2) we discuss free-swimming experiments in which we measured steady-state swimming speed, power consumption and calculated cost of transport for combinations of frequencies, amplitudes and wavenumbers in both the single- and double-finned robots. Our study expands the current understanding by examining how fin kinematics and fin shape affect swimming speed and energetics by building a four-dimensional parameter space that includes not only fin beat frequency and amplitude, but also wavenumber and fin shape.

## 2. Materials and Methods

This section describes the mechanical and control design of a single- and double-finned robot, free-swimming experiments and calculated variables—swimming speed, power consumption and cost of transport. While there are a wide range of measures to characterize swimming performance [25,26,27], we opted to use swimming speed and cost of transport in order to facilitate comparisons with previous studies because both measures are commonly used to characterize the performance of both animal and robotic swimmers [24,28,29,30,31,32,33,34].

### 2.1. Single-Finned Robot—Mechanical Design

**Figure 1:**
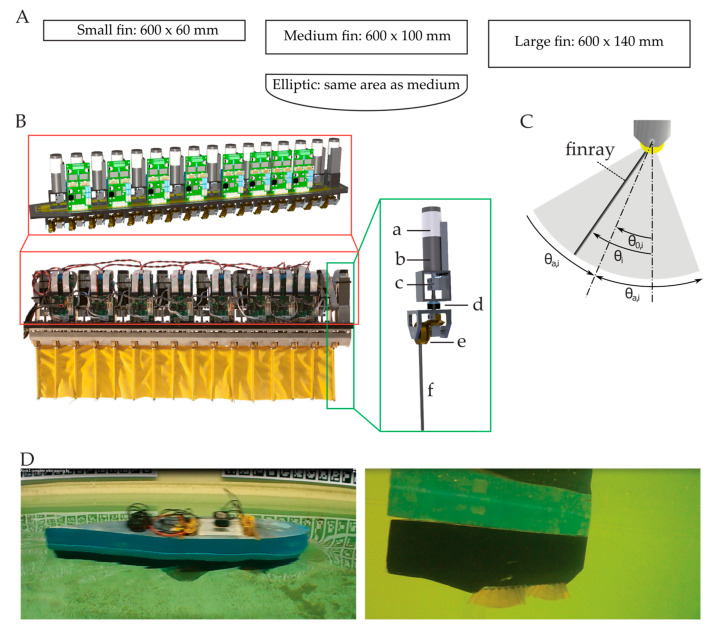
Robot and fin design. (**A**) Four fin designs. (**B**) Fin mechanism with 16 fin rays, waterproof cover removed. Each fin ray (f) was driven by its own actuator. The oscillatory motion of the motor (a) is transferred directly to the fin rays through a planetary gearbox (b), with a gearbox ratio of 1/53 and a 90° bevel gear combination (e), coupling (c) and oil seal (d). (**C**) *θ_i_*: angular deflection; *θ_a,i_*: angular amplitude; *θ*_0_*_,i_*: offset angle. (**D**) Stills of movies of the single-finned robot during forward swimming. Left: above-water view. Right: underwater view. Movies in Supplementary Materials.

Four different fin designs were studied: 600 × 140 mm (length × height, large fin), 600 × 100 mm (medium fin), 600 × 60 mm (small fin) and an elliptically shaped fin with the same surface as the medium fin (Figure 1A; see Appendix A, Table A2 for a list of experimental settings). All fins consisted of a single layer of Lycra–polyamide. The Lycra–polyamide has been used in previous mechanisms [14], and has a similar Young’s modulus as the membrane of a fish fin [15]. The membrane was attached to 16 fin rays, which resulted in a ray spacing of 40 mm.

Each fin ray was driven by its own actuator, a brushed DC motor (Maxon Re 24, 11 W) (Figure 1B). The actuators were driven locally by a custom-built motor controller board. The chosen configuration has a high range of angular deflection and the perpendicular rotating axis facilitated waterproofing. Separating the units facilitated assembly and maintenance. The fin ray–actuator combinations were mounted at a base, which functioned as a general support structure. The base and a carbon-fiber-reinforced plastic cover formed a watertight compartment to protect the electronics. The housing of the fin was covered by a streamlined body constructed from laminated carbon fibers. The housing’s shape was based upon an FX-71-L-150/20 airfoil with a length of 1.2 m and a width of 0.18 m at the thickest location [5]. Above the fin compartment, the central processing unit (CPU) and batteries were mounted on Styrofoam for buoyancy (Figure 1D). The Styrofoam had the same shape as the carbon body. The CPU was used to control the fin and log the signals from the different sensors. The CPU communicated wirelessly with a laptop to control the fin motion. Water could enter between the hull and the watertight fin compartment.

### 2.2. Single-Finned Robot—Control

The fin ray actuators were driven locally by a custom-built motor controller board, which has been used in several robots developed at the Delft Biorobotics Laboratory [35,36]. Each board could drive two brushed DC motors using a 1 kHz PID controller. The controller used the encoders mounted on the motor as a position feedback signal. As a secondary function, the board returned the applied voltage and current of each motor to evaluate the power consumption. The eight motor controller units communicated with a central processing unit through a serial communication bus. An undulating fin, constructed as a membrane supported by ridged fin rays, can be described as a discretely ruled surface [37], in which the oscillatory motion of each fin ray can be described by:

(1) $$\theta_{i}\left(t\right)=\theta_{a,i}\;\sin\;\left(2\pi f_{i}t+\varphi_{i}\right)+\theta_{0,i},$$

where *θ_i_* describes the angular deflection, *θ_a,i_* the angular amplitude, *f_i_* the frequency, *φ*_i_ the phase angle and *θ*_0,*i*_ the offset angle of the *i*th fin ray (Figure 1C) (for a list of symbols, see Appendix A, Table A1). The various motion patterns, from traveling wave to standing wave, were generated by varying the phase shift between successive fin rays: $\Delta \varphi =\varphi_{i+1}-\varphi_{i}$. If the desired kinematic fin pattern is a travelling wave moving from the front to the trailing edge, the phase angle of a fin ray can be described by: $\varphi_{i}=\frac{2\pi }{\lambda }x_{i}=\frac{2\pi k}{l_{f}}x_{i}$, with fin length *l_f_*, wavenumber *k*, wavelength $\lambda =\frac{l_{f}}{k}$ and position of the fin ray along the fin base, *x_i_*. The set points of the sinusoidal trajectory of each fin ray were calculated at the local controller. The eight boards were synchronized to avoid drift between the local controllers. Due to the low-level trajectory generation, the function of the higher-level controller was limited to generating the kinematic variables for each fin ray: frequency, angular deflection and phase angle. The second function of the CPU was collecting the data of different sensors and actuators. The high-level controller and data logger were embedded in the Robot Operating System (ROS) to facilitate integration in the final underwater robot.

### 2.3. Double-Finned Robot—Mechanical Design

**Figure 2:**
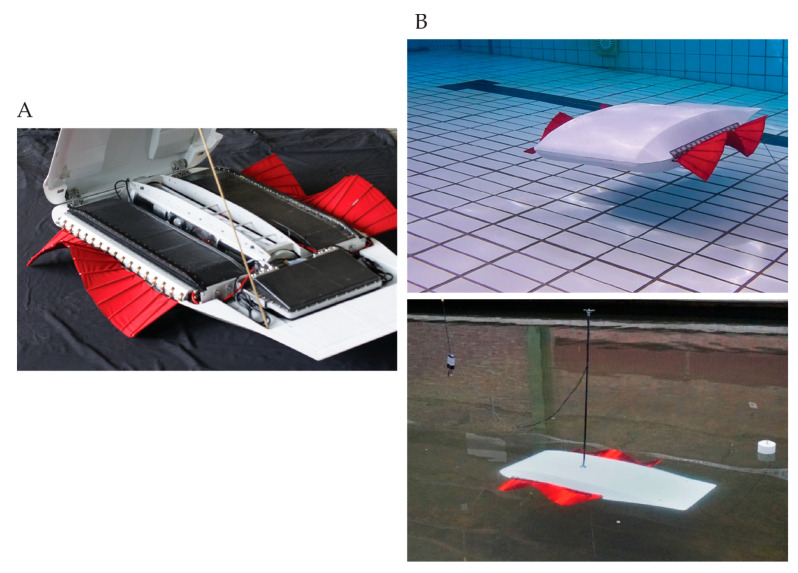
Double-finned undulatory robot. (**A**) Inside view of the double-finned robot. Watertight compartments at the sides housed the fin. A water-tight compartment at the rear housed the CPU. Buoyancy tanks were situated in the middle. (**B**) **Top**: remote-controlled swim tests in a pool. **Bottom**: remote-controlled free-swimming experiment in the shallow water basin at MARIN.

The double-finned robot consisted of eight watertight compartments that were attached to a support frame: two single-fin compartments as described in Section 2.1; a buoyancy compartment; two battery compartments; a pressure sensor compartment; a down-facing camera compartment; one CPU compartment (Figure 2A). The combined structure of compartments and frame was mounted within two streamlined bodies. The shape of the streamlined body was similar to the body used for the single-finned robot. The buoyancy compartment was a transparent tube in the center. It housed an inertia measurement unit (X-Sens^®^ Legacy mTi) and two modified Engel piston tanks. The two piston tanks allowed for trimming and making small adjustments in the pitch angle of the robot. The buoyancy tanks were mainly used to switch between clearly floating at the surface or being neutrally buoyant in the water. A battery compartment was mounted on each side of the buoyancy compartment. Each battery compartment housed four units of two Lipo cells (lithium-ion-polymer-accu), connected in series (28 V, 8 Ah). Each of the battery compartments supplied the power to one fin compartment and an auxiliary system, such as the buoyancy system, sensors and CPU. Below the buoyancy compartment, two sensors were mounted: a pressure sensor (Keller PAA-33X) and a down-facing camera. The rear compartment housed the CPU, which controlled all the subsystems and logged the signals from the different sensors. Using a floating antenna, the CPU of the robot communicated wirelessly with a laptop to control the robot. It worked similarly to the single-finned robot. The Lycra–polyamide fins had similar dimensions to the large fin on the single-finned robot (600 × 140 mm). Its dimensions were: 1.2 × 0.6 × 0.18 (length × width × biggest height). 

### 2.4. Experiments

#### 2.4.1. Accuracy of Fin Kinematics

We validated the accuracy of the fin motion by comparing prescribed and recorded kinematics for two cases in which only frequency was altered. The fin mechanism, equipped with a membrane of 105 × 600 mm, was submerged in an aquarium of 1.0 × 0.4 × 0.4 m (length, width, height). To derive the fin kinematics, the trajectory of each fin ray was recorded using the built-in encoders, attached to the motor. The fin ray positions were sampled with a frequency of 100 Hz. Based on these fin ray trajectories, the actual kinematic parameters—frequency, amplitude and phase angle—and their standard deviations were calculated over 750 cycles. The phase difference was calculated when the fin ray crossed the vertical axis (with an angular deflection of zero degrees), because the calculated phase difference depended on the angular deflection of the fin ray. At maximum deflection, the inaccuracy of the phase increased. Due to the resolution of the logged data (10^−3^ rad), the phase calculations were less precise at these points. The results of these validation tests are in Appendix B.

#### 2.4.2. Free-Swimming Measurements with a Single-Finned Robot

A single-fin free-swimming experiment was performed at the towing tank of the Faculty of Mechanical, Maritime and Material Engineering of the Delft University of Technology (Supplemental Material Video S2 and S3). The tank has a size of 50 × 2.75 × 1 m (length × width × depth). A total of 528 forward free-swimming runs were conducted along 30 m (4 fin shapes × 11 frequencies × 4 amplitudes × 3 wavenumbers; see Appendix A, Table A1). Frequencies ranged from 0.55 to 3.19 Hz in steps of 0.264 Hz, angular amplitude from 30° to 45° in steps of 5° and non-dimensional wavenumber from 1 to 2 in steps of 0.5 (fin length divided by wavelength).

To estimate the average velocity of the single fin in the towing tank, a vision-based system was used. A grid of fiducial markers was attached to each side of the towing tank. Two cameras were mounted on top of the fin for the localization of the robot in the tank. Both cameras were oriented to opposing walls of the towing tank and recorded images of 640 × 480 pixels at 20 Hz. Using the marker information, the relative displacement of the fin device was calculated using the ArUco library [38]. The average velocity was deduced from the relative displacement. Cost of transport was calculated as:

(2) $$\mathrm{COT}=\frac{P}{mv}$$

where *P* is the power consumption, *m* is the mass of the robot and *v* is the (steady) average swimming velocity. To estimate the power consumption of the entire fin, the voltage and current of each actuator was measured locally by the motor controller. The voltage and the current were logged with a resolution of 0.05 V and 0.02 A. The average consumed power of the entire fin was calculated as:

(3) $${\bar{P}}_{tot}=\bar{\sum_{i=1}^{16}P_{i}\left(t\right)}=\bar{\sum_{i=1}^{16}U_{i}\left(t\right)I_{i}\left(t\right)},$$

where *U_i_* is the local voltage and *I_i_* is the local current consumed by the *i*th fin ray. Free-swimming velocities, power consumption and cost of transport were linearly interpolated between measured points at new grid points for frequency, angular amplitude (MATLAB’s routine ‘meshgrid’ combined with ‘interp2’).

#### 2.4.3. Free-Swimming Measurements with a Double-Finned Robot

Free-swimming measurements with a double-finned robot were conducted at the shallow water basin at the Maritime Research Institute Netherlands (MARIN) in Wageningen, The Netherlands (dimensions: 220 × 15.8 × 1.1 m, length × width × depth) (swimming movie in Supplemental Material S4 video S4). MARIN made its Concept Basin available for these concept tests free of charge. We executed 36 steady forward swimming maneuvers with 15 different combinations of frequency, amplitude and wavenumber.

The carriage of the shallow water basin at MARIN was equipped with an optical tracking system (Certus Optotrak, tracker on top of the robot can be seen in Figure 2B, bottom). At MARIN, the output of this tracking system was integrated with the motion controller of the carriage. This allowed for the measurement system to follow a free-moving object through the entire tank. To follow the double-finned robot, a target was mounted on top of the double-finned robot. The output of this system was used to estimate the average velocity of the robot and was coupled to the fin kinematics. If more than one run was conducted with the same prescribed input kinematic variables, the median of the free-swimming velocity and power consumption was used.

## 3. Results

For the analysis, the large fin was used as the base case against which all other fins were benchmarked. The elliptical fin had similar results to the medium fin for all measured variables across all prescribed fin kinematics and was, therefore, not included in the comparison (see Appendix C for data on elliptical fin). Gaps in the parameter space occurred when data points could not be measured or analysis criteria were not met, such as the robot not reaching a steady-state swimming speed. These gaps in the data are evident as white regions in the heatmap graphs.

### 3.1. Single-Finned Robot Performance

#### 3.1.1. Velocity

**Figure 3:**
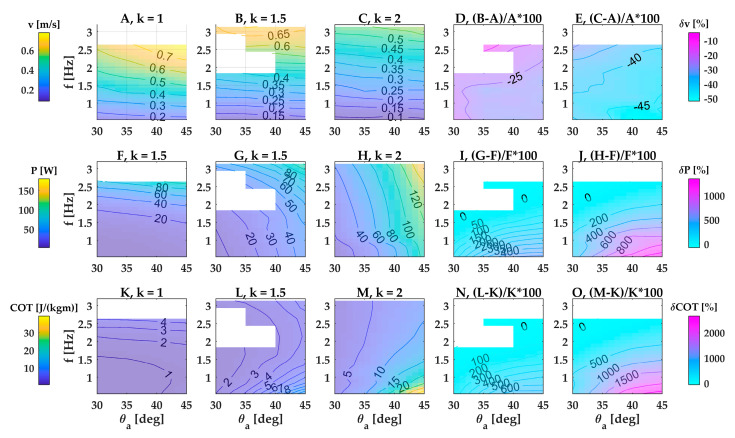
Speed, power consumption and cost of transport of a single-finned robot with fin dimensions 600 × 140 mm (large fin). Performance for three wavenumbers, plotted over angular amplitude and frequency: (**A**–**C**) free-swimming speed; (**F**–**H**) power consumption; (**K**–**M**) cost of transport. (**D**,**E**) Differences in free-swimming speed between *k* = 1.5 and *k* = 1 (**D**) and *k* = 2 and *k* = 1 (**E**), scaled by speed of panel A. (**I**,**J**) Differences in power consumption between *k* = 1.5 and *k* = 1 (**I**) and *k* = 2 and *k* = 1 (**J**), scaled by speed of panel F. (**N**,**O**) Differences in cost of transport between *k* = 1.5 and *k* = 1 (**N**) and *k* = 2 and *k* = 1 (**O**), scaled by cost of transport of panel K. The white regions indicate missing data.

**Figure A2:**
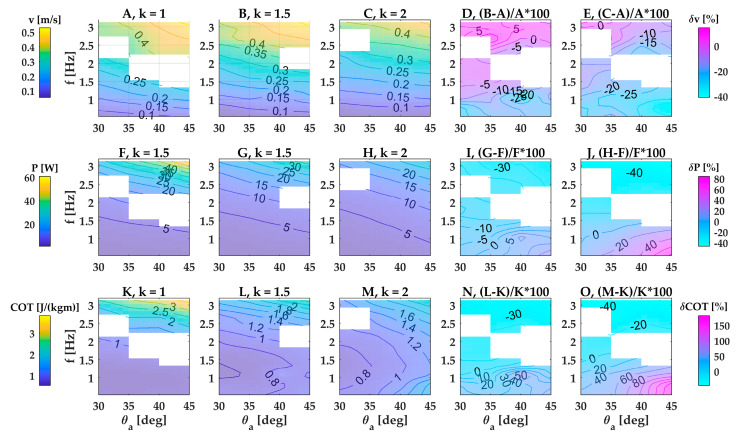
Speed, power consumption and cost of transport of a single-finned robot (fin dimensions 600 × 60 mm, small fin). Performance for three wavenumbers, plotted over angular amplitude and frequency: (**A**–**C**) free-swimming speed; (**F**–**H**) power consumption; (**K**–**M**) cost of transport. (**D**,**E**) Differences in speed between *k* = 1.5 and *k* = 1 (**D**) and *k* = 2 and *k* = 1 (**E**), scaled by speed of panel A. (**I**,**J**) Differences in power consumption between *k* = 1.5 and *k* = 1 (**I**) and *k* = 2 and *k* = 1 (**J**), scaled by speed of panel F. (**N**,**O**) Differences in cost of transport between *k* = 1.5 and *k* = 1 (**N**) and *k* = 2 and *k* = 1 (**O**), scaled by cost of transport of panel K. White regions indicate missing data.

**Figure A3:**
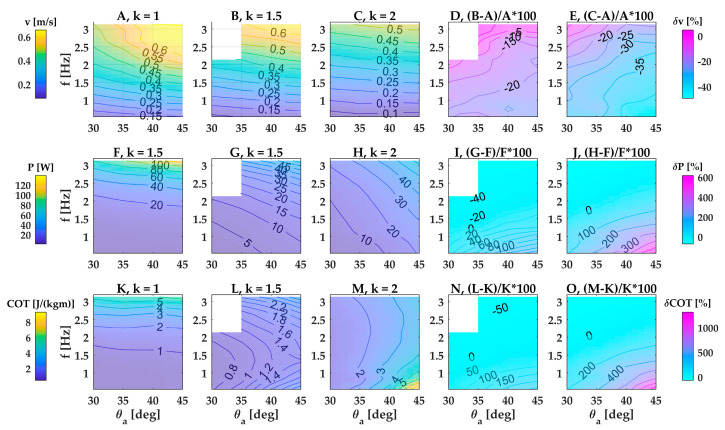
Speed, power consumption and cost of transport of a single-finned robot (fin dimensions 600 × 100 mm, medium fin). Performance for three wavenumbers, plotted over angular amplitude and frequency: (**A**–**C**) free-swimming speed; (**F**–**H**) power consumption; (**K**–**M**) cost of transport. (**K**–**M**) Cost of transport vs. amplitude and frequency for three wavenumbers. (**D**,**E**) Differences in speed between *k* = 1.5 and *k* = 1 (**D**) and *k* = 2 and *k* = 1 (**E**), scaled by speed of panel A. (**I**,**J**) Differences in power consumption between *k* = 1.5 and *k* = 1 (**I**) and *k* = 2 and *k* = 1 (**J**), scaled by velocity of panel F. (**N**,**O**) Differences in cost of transport between *k* = 1.5 and *k* = 1 (**N**) and *k* = 2 and *k* = 1 (**O**), scaled by cost of transport of panel K. White regions indicate missing data.

**Figure A4:**
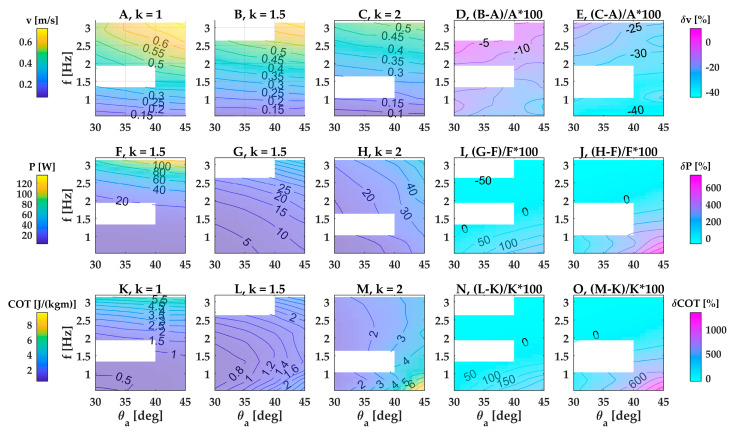
Speed, power consumption and cost of transport of a single-finned robot with elliptical fin that has the same area as the medium fin. Performance for three wavenumbers, plotted over angular amplitude and frequency: (**A**–**C**) free-swimming speed; (**F**–**H**) power consumption; (**K**–**M**) cost of transport. (**D**,**E**) Differences in speed between *k* = 1.5 and *k* = 1 (**D**) and *k* = 2 and *k* = 1 (**E**), scaled by speed of panel A. (**I**,**J**) Differences in power consumption between *k* = 1.5 and *k* = 1 (**I**) and *k* = 2 and *k* = 1 (**J**), scaled by speed of panel F. (**N**,**O**) Differences in cost of transport between *k* = 1.5 and *k* = 1 (**N**) and *k* = 2 and *k* = 1 (**O**), scaled by cost of transport of panel K. White regions indicate missing data.

In general, swimming velocity increased with increasing frequency and decreasing wavenumber across all four fins; of the three fin beat parameters we studied, angular amplitude had the weakest effect on swimming speed (Figure 3A–C; Figure A2, Figure A3 and Figure A4A–C).

In the base case of the large fin, the robot reached its highest swimming speed of 0.78 m/s (1.3 fin lengths per second or 0.65 robot lengths per second) at the highest fin beat frequency and amplitude and the lowest wavenumber (Figure 3A at *f* = 2.64 Hz, *θ* = 45°, *k* = 1). Swimming speed was most strongly modulated by wave frequency and wavenumber, whereas wave amplitude had only a small effect (Figure 3A–E). The three kinematic parameters interacted with each other, and the strength of the interaction changed across the parameter space. The weak effect of amplitude on swimming speed was modulated slightly by frequency and wavenumber (Figure 3A–E). In contrast, the strong effect of frequency on swimming speed was considerably modulated by wavenumber: increasing wavenumber by 50% from 1 to 1.5 caused a drop in swimming speed that ranged almost threefold from 10% to 27% across the examined frequency range (Figure 3D). Doubling wavenumber from 1 to 2 caused a unproportionally stronger drop in swimming speed by 29% versus 48% at high versus low frequencies (Figure 3E). Thus, within the examined parameter space, swimming speed increased with a lower wavenumber, and the combination of lowest wavenumber and highest frequency generated the highest swimming speed.

**Figure A5:**
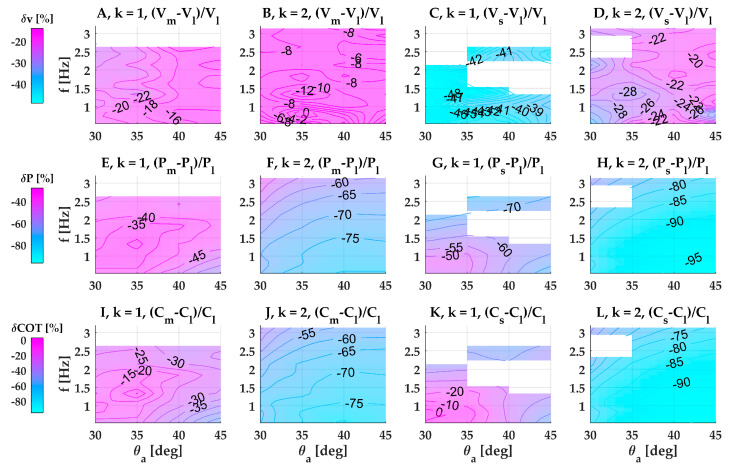
Differences in speed, power consumption and cost of transport of a single-finned robot for different fin dimensions for two wavenumbers, *k* = 1 and *k* = 2. In the titles of graphs I–L, cost of transport is abbreviated to C instead of COT for display reasons. Subscripts s, m and l stand for small, medium and large, respectively. (**A**–**D**) Difference in swimming speed between the medium and large fin for *k* = 1 and *k* = 2 (**A**,**B**) and small and large fin for *k* = 1 and *k* = 2 (**C**,**D**). (**E**–**H**) Difference in power consumption between the medium and large fin for *k* = 1 and *k* = 2 (**E**,**F**) and small and large fin for *k* = 1 and *k* = 2 (**G**,**H**). (**I**–**L**) Difference in cost of transport between the medium and large fin for *k* = 1 and *k* = 2 (**I**,**J**) and small and large fin for *k* = 1 and *k* = 2 (**K**,**L**).

The trends observed in the large fin were qualitatively similar in the medium and small fin (Appendix C, Figure A2, Figure A3 and Figure A4). The interaction between the three kinematic parameters, however, was stronger than in the large fin: swimming speed was more strongly modulated by frequency and amplitude at the lowest wavenumber, and this modulation weakened considerably as wavenumber increased, approaching the effect size observed in the large fin. Swimming speed decreased with decreasing fin height across all frequencies and amplitudes and the decrease was more pronounced at lower wavenumbers (Figure A5A–D).

Overall, smaller fins achieved lower swimming speeds, and swimming speed became less sensitive to undulation kinematics (frequency, amplitude, wavenumber) with decreasing fin height. Comparing the large fin with the two smaller fins, the medium fin achieved a speed that was up to 28.7% lower (at *f* = 2.93 Hz, *θ* = 30°, *k* = 1, Figure A5A), and small fin’s speed was up to 47.8% lower (at *f* = 1.63 Hz, *θ* = 30°, *k* = 1, Figure A5C).

#### 3.1.2. Power Consumption

In general, power consumption increased with increasing frequency and amplitude (Figure 3F–H; Figure A2, Figure A3 and Figure A4F–H). 

In the base case of the large fin, power consumption was lowest at the lowest low frequency, amplitude and wavenumber (Figure 3F–H) and reached an overall maximum of 159.3 W (Figure 3H) at the highest wavenumber, amplitude and frequency within our parameter space. The sensitivity of power to the three kinematic parameters (frequency, amplitude, wavenumber) changed across the parameter space. Frequency is a stronger modulator of power than amplitude at the lowest wavenumber, whereas the reverse is true at the highest wavenumber. Increasing the wavenumber by 50% from 1 to 1.5 caused changes in power consumption that ranged from a moderate decrease (−51.6% at *f* = 2.6 Hz, *θ* = 30°) to a considerable increase (+516.7% at *f* = 0.53 Hz, *θ* = 45°) (Figure 3I). Doubling the wavenumber from 1 to 2 caused an even wider range of changes in power consumption, from a similarly moderate decrease (−58.9% at *f* = 2.6 Hz, *θ* = 30°) to a steep increase (+1338% at *f* = 0.53 Hz, *θ* = 45°) (Figure 3J).

For the medium fin, as was the case for the large fin, frequency was a stronger modulator of power than amplitude at the lowest wavenumber, whereas the reverse was true at the highest wavenumber (Figure A3F–H). Increasing the wavenumber led to less power consumption at high frequencies and increased power consumption at low frequencies and high amplitudes, but to a lesser extent than with the large fin (Figure A3I–J). For the small fin, frequency was a stronger modulator of power than amplitude at all tested wavenumbers (Figure A3F–H). Again, increasing the wavenumber led to lower power consumption at high frequencies and higher power consumption at low frequencies and high amplitudes, but differences were small compared to the large and medium fin.

Power consumption decreased with decreasing fin height across all frequencies and amplitudes and increased with increasing wavenumbers (Figure A5E–H). In the large fin, increasing the wavenumber profoundly increased the power consumption. In contrast, in the small fin, wavenumber had a much weaker effect on power. This stronger effect of wavenumber led to large differences in peak power consumption between the three sizes of fin: at wavenumber *k* = 2, the large fin had an 80.4% higher power consumption than the medium fin (at *f* = 0.53 Hz, *θ* = 40–45°, Figure A5F), and a 96.6% higher power consumption than the small fin (at *f* = 0.53 Hz, *θ* = 45°, Figure A5H).

#### 3.1.3. Cost of Transport

Compared with swimming speed and power consumption, cost of transport ranged the most widely across the parameter space mapped in this study, changing by more than 2000% in the case of the large fin. Cost of transport also showed the most complex relation with the three kinematic parameters, frequency, amplitude and wavenumber. In particular, the location of peaks and valleys within the parameter space changed drastically with wavenumber.

In the base case of the large fin, wavenumber strongly affected how cost of transport changed with amplitude and frequency. At the lowest wavenumber (*k* = 1), frequency had a stronger effect than amplitude and cost of transport peaked at the highest frequency and amplitude. At the highest wavenumber (*k* = 2), amplitude had a stronger effect than frequency and the cost of transport peaked at the lowest frequency and the highest amplitude. Operating at an intermediate wavenumber (*k* = 1.5) caused peaks in cost of transport at both the lowest and the highest frequencies. In other words, to operate at low cost of transport, the large fin should employ a low frequency, amplitude and wavenumber. Beating at higher wavenumbers and amplitudes drastically increased costs of transport, especially at low frequencies. For example, doubling the wavenumber while beating at the lowest frequency and highest amplitude increased cost of transport by 2651% (Figure 3O).

In the medium and small fin, the complex relation between frequency and cost of transport persisted (Figure A2 and Figure A3K–M). When wavenumber was low, cost of transport was lowest at low frequencies and was quite insensitive to amplitude. When wavenumbers were intermediate or high, cost of transport became less sensitive to frequency and more sensitive to amplitude and was lowest at intermediate frequencies. Increasing the wavenumber caused steep increases in cost of transport of up to 1312%, but less severely than in the large fin (Figure A2 and Figure A3N,O). Cost of transport decreased with decreasing fin height across all frequencies and amplitudes and the difference was higher at higher wavenumbers (Figure A5I–L).

Overall, the following trends emerged. Swimming speed was more strongly affected by frequency than amplitude across the wavenumbers and fin heights examined in this study. Power consumption was sensitive to frequency at low wavenumbers, and increasingly sensitive to amplitude at high wavenumbers. This increasing sensitivity of amplitude was modulated by fin height; it was pronounced in the large fin, and weak in the small fin. Cost of transport had the most complex relation with fin size and kinematics and changed the most drastically across the parameter space mapped in this study. Both power consumption and cost of transport were increasingly affected by amplitude as fin height increased (Figure A5).

### 3.2. Velocity, Power Consumption and Cost of Transport of the Double-Finned Robot

**Figure 4:**
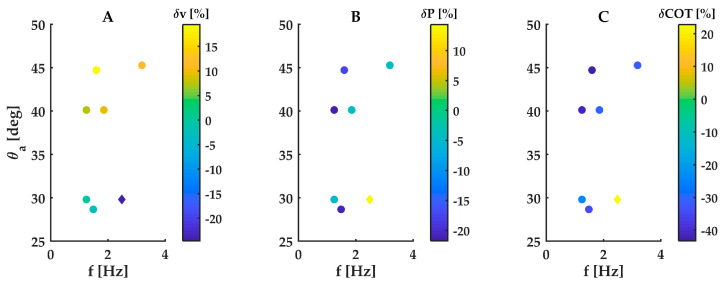
Difference in speed, power consumption and cost of transport between a double- and single-finned robot at equal fin kinematics (reference case is the single-finned robot). (**A**) Swimming velocity difference. (**B**) Power consumption difference. (**C**) Cost of transport difference. Round data points are k = 1.5, diamond data point is k = 1.

**Figure A6:**
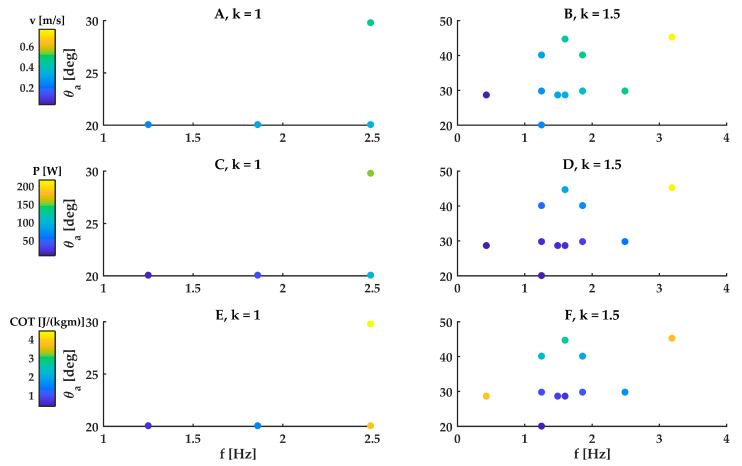
Speed, power consumption and cost of transport of a double-finned robot. (**A**,**B**) Free-swimming speed vs. amplitude and frequency for two wavenumbers. (**C**,**D**) Power consumption vs. amplitude and frequency for two wavenumbers. (**E**,**F**) Cost of transport vs. amplitude and frequency for two wavenumbers.

The comparison between the single- and double-finned robots is confounded by the fact that the two robots have different drags due to differences in their position in the water column: the double-finned robot was totally submerged with only an antenna sticking out, while the single-finned robot swam with part of the hull breaching the surface. Given this caution, the trends observed in the double-finned robot qualitatively resembled those observed in the single-finned robot: swimming speed increased with increasing wavenumber, amplitude and frequency (Appendix D, Figure A6). Yet the double-finned robot did not consistently outperform the single-finned robot. The double-finned robot underperformed at the lowest wavenumber (*k* = 1), swimming at a 25% lower speed (0.46 m/s) than the single-finned robot (0.61 m/s) at the same fin kinematics (*f* = 2.5 Hz, *θ* = 29.7°) (Figure 4A). At the higher wavenumber (*k* = 1.5), the double-finned robot caught up and began to outperform the single-finned robot as amplitudes and frequencies increased. At its peak speed (0.76 m/s), it outperformed the single-finned robot by 13% (0.67 m/s; *f* = 3.19 Hz, *θ* = 45.3°) (Figure 4A).

Power consumption also largely followed the trends observed in the single-finned robot, increasing with increasing speed and peaking at 153 W (214 W) for *k* = 1 (*k* = 1.5) (Appendix D, Figure A6). At the same fin kinematics, the double-finned robot generally consumed slightly less power than the single-finned robot for k = 1.5 and slightly more for k = 1 (Figure 4B). Yet its power consumption increased more steeply with increasing wavenumber, frequency and amplitude, causing it to be outperformed by the single-finned robot at its peak power consumption of 153 W (*k* = 1) and 214 W (*k* = 1.5). At the same kinematics as those two incidences of peak power, the double-finned robot’s power consumption was roughly double that of the single-finned robot (153 W vs. 67 W at *k* = 1, 214 W vs. 112 W at *k* = 1.5).

Cost of transport also largely followed the trends observed in the single-finned robot. Again, wavenumber had a strong effect on the shape of the performance landscape. At the same fin kinematics, the double-finned robot generally consumed less power than the single-finned robot for k = 1.5 and slightly more for k = 1 (Figure 4C). At the low wavenumber of *k* = 1, cost of transport generally increased with increasing frequency and amplitude to a maximum of 4.4 J/kg m (*f* = 2.5 Hz, *θ* = 29.7°, Appendix D, Figure A6E). In contrast, at the high wavenumber of *k* = 1.5, the double-finned robot reached a high COT of 3.85 J/kg m at a low frequency (*f* = 0.43 Hz, *θ* = 28.6°, Appendix D, Figure A6F) and very low swimming speed (0.03 m/s). Compared with the single-finned robot at the same kinematics, the double-finned robot had a higher cost of transport at the lower wavenumber (4.4 J/kg m vs. 3.6 J/kg m at *k* = 1, *f* = 2.5 Hz, *θ* = 29.7°), but a lower cost of transport at the higher wavenumber (3.85 J/kg m vs. 5.5 J/kg m at k = 1.5, *f* = 0.43 Hz, *θ* = 28.6°). At its maximum swimming speed, the double-finned robot’s cost of transport was 3.78 J/kg m (f = 3.19 Hz, θ = 45.3°, Appendix D, Figure A6F).

## 4. Discussion

In this study, we designed, developed and constructed a single-finned and double-finned robot. We ran forward free-swimming experiments with four different fin designs (3 different heights and one different overall shape, elliptic). We measured free-swimming forward speed, power consumption and cost of transport for a range of fin-flapping frequencies, angular amplitudes, wavenumbers and fin height for both robots. Our study demonstrated the power of robotic models to examine the relation between fin kinematics and swimming performance beyond the parameter space inhabited by animal swimmers. In the following comparison with animal swimmers and industry-grade robots, we show that biomimetic robotic swimmers can compete with animal swimmers in terms of power requirements, but they do not outperform industry-grade AUV designs [39].

Our four main findings are as follows. First, swimming speed increased with increasing frequency, amplitude and decreasing wavenumber across all fins. This combination of kinematic variables gave a combination of the highest backward travelling wave speed (*v*_wave_ = *fλ*) with high amplitude (so large backward travelling fin surface). 

Second, power consumption increased with increasing frequency, amplitude and wavenumber for the large fin. With decreasing fin height (medium and small fin), power consumption increased with increasing frequency, amplitude and decreasing wavenumber. The increase in power consumption with increasing frequency suggests that more power was needed to accelerate and decelerate the motor during higher frequencies. The increase in power consumption with increasing fin heights could be caused by fluid dynamic (such as changes in vortex-shedding dynamics) or solid mechanic effects (the higher amplitude and wavenumber causes a large phase angle between consecutive fin rays, requiring more power to stretch the viscoelastic fin material).

Third, for the large fin, COT increased with decreasing frequency and increasing amplitude and wavenumber. For the medium and small fin, COT increased with increasing frequencies at a low wavenumber (*k* = 1) and increasing amplitude at a high wavenumber (*k* = 2). Fourth, swimming velocity, power consumption and cost of transport at equal fin kinematics all decreased with decreasing fin height. Furthermore, we observed that the robot heaved up and down at low wavenumbers. This observation suggests that at higher wavenumbers, the fin imparts more backward than vertical momentum, leading to a steadier motion, as also noted in [24].

### 4.1. Comparing Kinematics and Swimming Speed among Robotic and Animal Swimmers

The robots in this study used fin kinematics that overlap with values found in animal swimmers, resulting in comparable performance and performance trends. The single-finned robot used a range of fin beat frequencies, fin amplitudes and wavenumbers that overlapped with values observed in stingrays, ghost knifefish and small skates (Table 1). The robot’s performance overlaps with that of animal swimmers [40,41,42,43,44], and it shows similar relations between fin kinematics and performance, such as small skates enhancing thrust by increasing wave speed and frequency but not amplitude [44].

The robots in this study performed within the top 15% fastest fish robots as reviewed by White, Lauder and Bart-Smith [46], most of which are body-caudal-fin-propelled robots. Additionally, the trends observed in this study confirm previous studies with other finned robots, such as swimming speed increasing with increasing frequency, and speed decreasing when wavenumber is lowered [24].

### 4.2. Comparing Power and Cost of Transport among Robotic and Animal Swimmers

**Figure 5:**
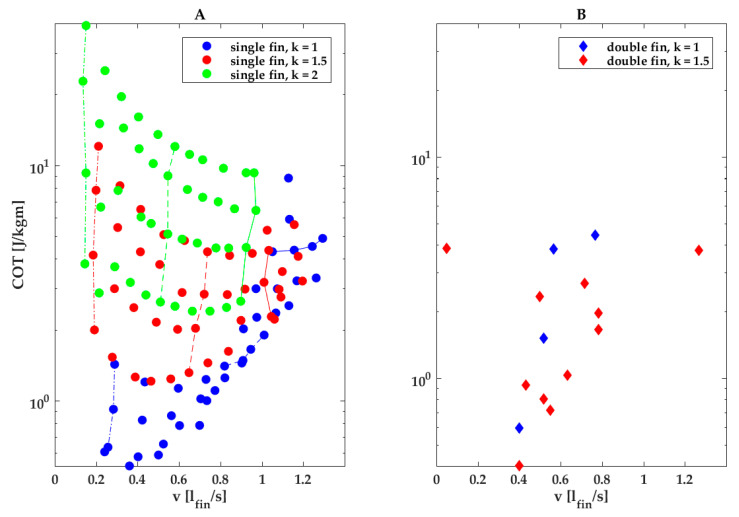
COT vs. fin length normalized swimming speed for a single-fin robot (**A**) and a double-fin robot (**B**) for different wavenumbers. (**A**,**B**) Green represents *k* = 2, red *k* = 1.5 and blue *k* = 1. (**A**) Dash-dotted line with full circles for all colors indicates COT values for f = 0.53 Hz. Dotted line with full circles for all colors indicates COT values for *f* = 1.86 Hz. Solid line with full circles for all green and red indicates COT values for *f* = 3.13 Hz and for blue 2.67 Hz (no data for 3.13 Hz). Lowest placed circles for each vertical line indicate COT values for angular amplitude of 30°. Highest placed circles for each vertical line indicate COT values for angular amplitude of 45°. The values in between are for 35° and 40° amplitude.

Our parametric study observed the U-shaped COT vs. speed curves predicted by hydrodynamic theory and observed in fish, with minima at intermediate swimming speeds and higher energy expenditure at lower and higher swimming speeds (Figure 5) [47]. Our study further finds that in our finned robot, COT is minimal near the transition from oscillating to undulating fin kinematics (Figure 5, blue dots; wavenumber *k* near or just below 1). The location of the minimum shifts with wavenumber and angular amplitude; the COT-over-speed curve showed a clear U shape at low angular amplitudes and low wavenumbers, but the minimum shifts to higher speeds at higher amplitudes and wavenumbers beyond the parameter space examined in our study. 

Amplitude was a strong driver of COT, especially at high wavenumbers, as evident in Figure 5A. In Figure 5A, the lines connecting data points taken at the same wavenumber and frequency yet increasing amplitude are nearly vertical for the highest wavenumber (Figure 5A, green lines and dots), indicating that increasing the amplitude does not increase swimming speed. In contrast, at low wavenumbers (Figure 5A, blue lines and dots), increasing the amplitude does increase swimming speed, especially at the higher frequencies, as evident in the blue lines tilting increasingly to the right, which indicates that an increase in amplitude at high frequencies and low wavenumbers has only a small effect on COT (Figure 5A, blue lines and dots). We speculate that a high angular amplitude drives up COT at higher wavenumbers (*k* = 1.5 and *k* = 2; Figure 3K–M) due to the increasing cost of deforming the fin membranes.

The double-finned robot was not more energy efficient than the single-finned robot at comparable fin kinematics. Their COTs were similar at comparable fin normalized swimming speeds, although half of the measurement points at wavenumber *k* = 1.5 lie in the *k* = 1 region for the single fin (Figure 5B).

The biological studies that we used for the comparison with our robot only measured the decline in COT with increasing swimming speed (so the left part of the U curve approaching the optimal cruising speed) [40,41,42,43,44], as did several other biological studies on swimmers using fin or body undulations [48,49,50]. For example, COT in small skates decreases from 15 to 5 J/(kg m) as swimming speed increases from 0.75 to 1.25 BL [50]; COT in *Loligo* drops from 60 to 10 J/(kg m) as swimming speed increases from 0.5 to 5 mantle length/s [49]; COT in catfish decreases from 80 to 5 J/(kg m) as swimming speed increases from 0.2 to 2 BL/s [49].

Other studies on fish-like robots observed similar trends: a review of fish-like robots propelled by a caudal fin and found a similar U-shape curve for COT over swimming speed [46]. Although a direct comparison among robots is difficult, comparing the robots from the aforementioned review with our single- and double-finned robot COT at their maximum swimming speeds (4.8 J/kg m single-finned robot @1.29 fin lengths per second or 0.78 m/s; 3.8 J/kg m double-finned robot @1.26 fin lengths per second or 0.76 m/s, Figure 5A,B) would place our robots in the top 3 most efficient out of the 15.

This study focused on cost of transport as a measure of locomotion efficiency. Here, cost of transport is calculated based on the power consumption for propulsion. In reality, other subsystems such as CPU, communication and data transfer also require power, often referred to as ‘hotel power consumption’ [39]. We did not specifically measure our hotel consumption, but a first educated guess would be around 20 W. That implies that the cost of transport would increase by 15 to 100% for the large fin and 30 to 400% for the small fin, over the kinematic parameter space measured. Thus, the presented fin-related cost of transport numbers are an underestimation of the total costs. It is important to mention that subsystems were never optimized for power consumption.

A comparison with industry-grade robots revealed that aquatic animals typically have a higher COT than engineered systems of equivalent size when COT includes not only propulsion but all costs [39]. In animals, that cost is based on metabolic cost and includes resting metabolic rate; in engineered systems that cost includes so-called hotel costs, that is, the power needed for all subsystems besides propulsive power. Compared with those industry-grade robots, our finned robots’ COT is in the same range as those AUVs, and the highest COT values of our robots are similar to those of marine mammals.

While cost of transport (COT) is a good efficiency metric for robots that travel large distances, it might not be the best to investigate the efficiency of robots that are inspired by animals designed to excel in maneuvering in tight spaces, such as knifefish [1,51], cuttlefish and squid [2,3]. It would be interesting to look at cost of maneuverability or cost of stability. Cost of maneuverability can be approached by measuring the power consumption during yaw, pitch and roll maneuvers at different turning radii, velocities and accelerations. From a survey and inspection perspective, cost of stability is an interesting one. The more stable a survey or inspection robot is, the easier the piloting (if remotely piloted) and the less motion correction post-processing on survey data needs to be performed. Cost of stability is then not only a pure power-input-to-output metric, but it also involves operational cost of stability, i.e., cost of extra post-processing and cost of challenging piloting, including the cost of training pilots.

This study described only steady forward swimming, as it is the least complex maneuver to analyze. Recorded swimming experiments with the double-finned robot at MARIN included several yaw, pitch, roll, hover and turning on the spot maneuvers. Those experiments remain to be analyzed to give an indication of cost of maneuverability or stability. Additionally, the fin is capable of generating standing waves to execute lateral excursions and oscillations with linearly increasing and decreasing amplitude.

## Figures and Tables

**Table 1:** Kinematic parameters of robotic and animal swimmers using undulating fins.

Swimmer	Frequency (Hz)	Amplitude	Wavenumber	v_max_ (L/s) ^1^
robot	0.55–3.19	30°–45°	1–2	0.65 RL/s 1.3 FL/s
stingray	1.5–3.0 ^2^ 0.78–4.2 ^3^	0.21 (mid disk) DL ^2^ 0.08–0.35 DL ^3^	— 0.4–1.31 ^3^	— 0.9–3 DL/s ^4^
knifefish	6.74 ^5^ 5.1–9.3 ^6^	— 70° (max) ^6^	3.38 ^5^ —	0.51 BL/s ^5^ 0.18–2.0 BL/s ^6^

^1^ normalized swimming speed using the swimmer-appropriate length: RL—robot length, FL—fin length, DL—disk length, BL—body length; ^2^ Blevins and Lauder 2012 [40]; ^3^ Rosenberger 2001 [41]; ^4^ Rosenberger and Westneat, 1999 [42]; ^5^ Youngerman, Flammang and Lauder, 2014 [43]; ^6^ Ruiz-Torres et al., 2013 [45].

**Figure A1:**
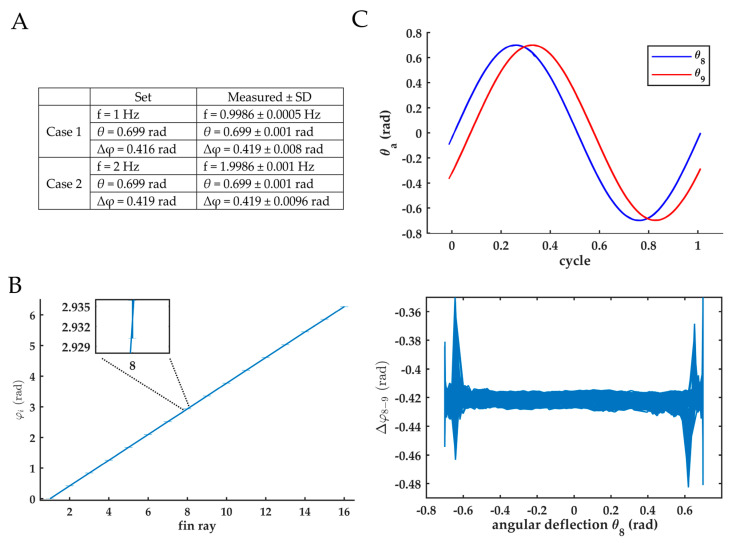
Verification of fin kinematics. (**A**) Two kinematics used to evaluate fin ray performance. Column ‘set’: prescribed kinematics; Column ‘measured ± SD’: observed parameters of the motion of 9th fin ray, while submerged. Mean and standard deviation were calculated over 750 cycles. (**B**) The observed phase angle of each fin ray. Error bars indicate standard deviation calculated over 750 cycles (case 1). (**C**) Top: sinusoidal trajectory of the angular deviation the 8th and 9th fin ray are shown. The 750 cycles are mapped on top of each other (case 1). Both lines show a high repeatability and a constant phase shift. Bottom: phase shift between the 8th and 9th fin ray versus the deflection angle.

## Data Availability

Data are available upon request to the corresponding author.

## Supplementary Materials

The following supporting information can be downloaded at: https://www.mdpi.com/article/10.3390/biomimetics8020214/s1, Video S1: Water jet along an undulating fin. Green and red color are iso-velocity surfaces at 1.2U∞(green) and 1.5U∞(red); Video S2: Test runs of a single-finned robot, showing the complete robot; Video S3: Test runs of a single-finned robot, showing fin movement; Video S4: double-finned robot swimming in a shallow water basin.

## Appendix A. Symbols and Experimental Parameters

Below is a list of symbols [units] and a Table detailing experimental settings.

**Table A1:** List of symbols [units].

Symbol	Definition	Unit
*Θ_i_*	Angular deflection	[deg]
*θ_a_*	Angular amplitude	[deg]
*θ*_0_	Offset angle	[deg]
*λ*	Wavelength	[mm]
*φ*	Phase angle	[deg]
COT	Cost of transport	[W/(kg m/s)]
*f*	Frequency	[Hz]
*i*	Fin ray number	[-]
*I*	Local current	[A]
*k*	Wavenumber	[-]
*l*_f_	Fin length	[mm]
*m*	Robot mass	[kg]
*P*	Power consumption	[W]
*U*	Local voltage	[V]
*v*	Steady average swimming speed	[m/s]
*x*	Position along fin base	[mm]

**Table A2:** Experimental parameters used in this study.

Parameter	Symbol	Values	Unit
fin height	—	60, 100, 140 mm	mm
fin beat frequency	*f*	0.55 to 3.19 Hz in steps of 0.264 Hz	Hz
fin beat amplitude	*θ_a_*	30° to 45° in steps of 5°	deg
wavenumber	*k*	1, 1.5, 2	—

## Appendix B. Accuracy of Fin Kinematics

Prescribed and recorded fin kinematics showed excellent similarity. Mean recorded frequency differed less than 0.15%, while mean recorded amplitude and phase were similar for two cases with different frequencies (A). The standard deviation of the phase difference between consecutive fin rays for case 1 was consistently small (B,C bottom). The motion of the 8th and 9th fin ray showed high repeatability (C, top).

## Appendix C. Free-Swimming Performance of Small, Medium and Elliptic Fin

The single-finned robot when mounted with the small, medium and elliptic fin performed as seen in the figures below.

## Appendix D. Velocity, Power and Cost of Transport of a Double-Finned Robot

The double-finned robot performed as seen in the figure below.
